# The reproducibility of self-reported age at menarche: The Tromsø Study

**DOI:** 10.1186/s12905-017-0420-0

**Published:** 2017-08-22

**Authors:** Marie Wasmuth Lundblad, Bjarne K. Jacobsen

**Affiliations:** Department of Community Medicine, UiT- The Arctic University of Norway, Tromsø, Norway

**Keywords:** Menarche, Reproducibility, Reliability, Menstruation, Correlation

## Abstract

**Background:**

Previous studies of the reproducibility of self-reported age at menarche have been limited because of small study samples, short follow-up and the limited age span of the women included.

**Methods:**

The present study assessed the reproducibility of age at menarche in 6731 women with a wide variation of age when giving the information about age at menarche. The women reported age at menarche in a self-administered questionnaire, both in 1986–1987 and 1994–1995. They were all residents of Tromsø, Norway, and aged 25–73 in 1994–1995. In order to investigate the agreement between self-reported age at menarche at the two points in time, Pearson’s correlation coefficient was applied to assess the linear correlation between the reported menarcheal age at the two occasions. Analyses were stratified for age. A Bland-Altman plot was produced and limits of agreement computed.

**Results:**

We found a high correlation and a strong agreement between self-reported age at menarche in 1986–1987 and 1994–1995. The overall Pearson’s correlation coefficient was 0.84 and was not attenuated by increasing age of the women. The Bland-Altman plot showed a strong agreement in self-reported age at menarche. The mean difference between self-reported age at menarche was 0.01 years with limits of agreement −1.52 to 1.54.

**Conclusion:**

We found high reproducibility of self-reported age at menarche. The mean menarcheal age in the two surveys was identical (13.2 years) with 95% of the women reporting the same age at menarche or with a difference of 1 year. Only 0.7% of the women reported age at menarche with a difference of more than 2 years in 1986–1987 and 1994–1995.

## Background

Menarche is the first menstrual bleeding, and it marks the beginning of reproductive life for women. It is a significant event, which has received considerable attention in reproductive epidemiology research. Studies have linked earlier age at menarche to higher morbidity and mortality later in life [1–3]. Furthermore, a trend toward a lower mean age at menarche has been observed in many countries, which may have public health implications [4] like higher all-cause mortality [2] and cardiovascular disease mortality [5–7], higher risk for breast cancer [8], cardiovascular disease [7, 9], diabetes [10–12], obesity [3] and metabolic syndrome [13, 14]. Early age at menarche has also been linked to psychological disorders, depression, smoking and alcohol use in adolescence and also early sexual behavior [15–17].

Mean age at menarche varies according to socioeconomic status, living conditions, ethnicity and several other factors, and differs therefore between populations in different parts of the world. Menarche usually occurs between ages 8 and 16 in developed countries, and the mean age at menarche has been reported to vary from 12.0 years in Italy to 13.5 years in Germany [18].

In order to evaluate and investigate time changes in age at menarche, and its association to the conditions mentioned above, information about the age when menarche occurs is necessary. The validity and reproducibility of self-reported menarche have been questioned, and a low validity and/or reproducibility of reported menarcheal age (how accurately women remember their menarcheal age and to what extent they give consistent information) will introduce misclassification in the analyses and produce biased estimates. It is challenging to ensure that the information regarding age at menarche is accurate without extensive monitoring. Most research therefore relies on self-reported menarcheal age or parental reported menarcheal age.

Most studies of the validity of self-reported age at menarche show a moderate to high validity (*r* = 0.66–0.83) when comparing actual age at menarche with recalled age at menarche later in life [19–21]. The sample sizes of the validity studies ranged from 132 to 1050 participants. Research focusing on the reproducibility of self-reported menarche shows a moderate [22–24] to strong [25–29] reproducibility of reported age at menarche at two different points in time. Only one study reports inconsistency in reported age at menarche when girls were asked repeatedly with one-year intervals. It was concluded that girls change their minds regarding when they experienced menarche as they grow older, and that self-reported menarche is therefore not reliable [30].

However, previous reproducibility studies have been limited because of relatively low number of participants (from 24 to 253) [22, 24–29], short time between recall of age at menarche (from 3 weeks to 3 years) [25–29] and age groups included (limited to adolescents or older women only) [22–24, 26, 27, 31]. The largest study published included 1976 girls (4th to 9th grade) followed for a maximum of 3 years [31]. Thus, there is a need for larger studies that provide the opportunity for assessing the reproducibility of women with different ages and with a relatively long period between the data collections in order to obtain more knowledge about the reproducibility of self-reported age at menarche.

The aim of the present study was to investigate the reproducibility of self-reported menarcheal age in a large group of women aged 25–73 years who had given the same information 7 years earlier. We also investigated whether the reproducibility was attenuated by increasing age of the women.

## Methods

The Tromsø Study, which is a prospective cohort study, was initiated in 1974 and is now one of the largest epidemiological studies in Norway [32]. Tromsø is the largest city in North Norway with a total of 72,681 inhabitants as of January 1st 2015 [32, 33]. The main aim of the Tromsø Study was to determine the reasons for the high cardiovascular mortality in Northern Norway in the 1970’s, and to find ways to prevent cardiovascular diseases.

A total of 7 surveys have been conducted, the last one in 2015–2016, and the Tromsø Study has been considerably expanded and now includes research of a number of topics relevant for chronic diseases. The first survey included only men, as cardiovascular diseases were considered mainly a problem for men, but in all the 6 later surveys, both men and women were invited. The design of the Tromsø Study is detailed elsewhere [32].

Briefly, the participants were invited to a physical examination with anthropometric measurement and blood samples and were asked to fill in questionnaires. A question about age at menarche was included for the first time in the third Tromsø Study survey (Tromsø 3), which was conducted in 1986–1987. The relevant question asked in the self-administered questionnaire was “How old were you when you started menstruating?” The women were asked to state how old they were in years as integers (e.g., 13 years). English translation of the questionnaires used in Tromsø 3 and Tromsø 4 are accessible (https://uit.no/Content/271763/T3_Q2.pdf) (https://uit.no/Content/430574/T4_Q2_U70.pdf).

All women in Tromsø aged 20 to 56 years were invited to take part in Tromsø 3 in 1986–87. A random sample of 10% in the age-group 12–19 years, in addition to the family members (women and children) of men who in Tromsø 2 (1979–1980) had been identified as being at high-risk for cardiovascular diseases, were also invited to participate [34]. A total of 10,863 women participated (79% of the 13,745 invited women) [32]. Tromsø 4, conducted in 1994–1995, is the largest of the Tromsø Study surveys. All residents of Tromsø older than 25 years of age were invited to the survey. Out of the 19,078 women who were invited, 14,293 women (75%) attended [32]. The attending women were given a self-administered questionnaire including the identical question regarding age at menarche.

A total of 6731 women answered the question regarding age at menarche in both Tromsø 3 and Tromsø 4, and were included in the present analyses. The women had no access to their first response when they completed the questionnaire 7 years later. The overall reproducibility of self-reported age at menarche was first assessed by the Pearson’s correlation coefficient between the age at menarche according to Tromsø 3 and Tromsø 4. The mean difference between the age at menarche stated in the two surveys was computed, as well as the percentage of the women who stated the same age and the percentage that indicated age at menarche with one, two, or more than two years difference in the two surveys. Stratified analyses were performed according to age groups in Tromsø 4: 25–34 years, 35–44 years, 45–54 years and 55–73 years. As there were only 65 women aged 65–73, this group of women was merged with women aged 55–64.

The correlation coefficient has some weaknesses when it comes to describing agreement between two measurements [35]. For the purpose of further investigating the agreement between self-reported age at menarche in Tromsø 3 and Tromsø 4, a Bland-Altman plot was included where the difference between age at menarche in Tromsø 3 and Tromsø 4 is indicated on the ordinate and the mean of the two menarcheal ages on the abscissa. The Bland-Altman plot is regarded as an appropriate method to apply when investigating the agreement as in the present context, and it provides useful information in addition to that conveyed by the correlation coefficient. The plot shows the agreement between information concerning age at menarche in Tromsø 3 and Tromsø 4 and includes the limits of agreement, which is calculated as the mean difference ± 1.96 times the standard deviation of the difference between recorded age at menarche in Tromsø 3 and 4. The limits of agreement display how dispersed reported age at menarche at two time-periods is likely to be for most (95%) of the women.

The analyses are based on the much larger Tromsø Study database, and each project based on it has to be authorized and data cannot be shared.

The SPSS statistics 22 package was used to analyze the data.

The Tromsø Study is approved by the Regional Committee for Medical Research Ethics. All included subjects provided written consent.

## Results

The study included 6731 women, who reported menarcheal age in both 1986–1987 (Tromsø 3) and 1994–1995 (Tromsø 4). Mean age in Tromsø 4 of the women included in the analyses was 45.3 years (standard deviation (SD): 9.9). In Tromsø 4, few women reported to be young (<11 years) or old (>15 years) at menarche, 1.5 and 4.7%, respectively. Self-reported age at menarche in Tromsø 4 ranged from 8 to 21 years. Mean age at menarche according to the information in Tromsø 3 and Tromsø 4 was 13.2 years (SD 1.28) and 13.2 (SD 1.30), respectively (Table 1). Thus, no systematic difference was present.

**Table 1:** The reproducibility of self-reported age at menarche according to age in Tromsø 4, among 6731 women participating in Tromsø 3 (1986–87) and Tromsø 4 (1994–95). Mean values and correlation coefficients. The Tromsø Study

		Self-reported age at menarche (years)				
Age (Tromsø 4)	N (%)	Tromsø 3	Tromsø 4	Difference (years)	Absolute difference (years)	Correlation coefficient
25–34	1058 (15.7)	13.04 (1.32)^a^	13.05 (1.35)	0.01 (0.83)	0.49 (0.67)	0.81
34–44	2154 (32.0)	13.07 (1.29)	13.11 (1.32)	0.04 (0.76)	0.44 (0.62)	0.83
45–54	2131 (31.7)	13.20 (1.32)	13.19 (1.38)	−0.01 (0.77)	0.43 (0.64)	0.84
55–73	1388 (20.6)	13.59 (1.40)	13.58 (1.37)	−0.01 (0.76)	0.42 (0.64)	0.85
Total	6731 (100.0)	13.21 (1.34)	13.22 (1.37)	0.01 (0.78)	0.44 (0.64)	0.84

^a^Mean (standard deviation)

Analysis showed a strong correlation between self-reported menarcheal age in Tromsø 3 and Tromsø 4 (Pearson’s *r* = 0.84, *P* < 0.001) (Table 1). This strong correlation held for all age groups and did not attenuate with increasing age of the women. On the contrary; among those in the youngest (25–34 year) age group, the Pearson’s *r* was 0.81, whereas it was highest in the oldest age group (55–73 years) (*r* = 0.85). Mean difference of reported menarcheal age in Tromsø 3 and Tromsø 4 was 0.01 (SD: 0.78). A mixed model analysis that took into account the repeated measures of age at menarche confirmed that the reported age at menarche did not differ between Tromsø 3 and Tromsø 4.

The mean absolute difference, in one direction or the other, was 0.44 (SD 0.64). The mean absolute difference ranged from 0.42 years in the oldest age group (55–73 years) to 0.49 years in the youngest age group (25–34 years) (Table 1).

Table 2 details the proportion of the women who reported their age at menarche with perfect concordance (the same year) and with a difference of one, two, or more than 2 years. In the youngest age group (25–34 years), 58.9% reported their menarcheal age in Tromsø 4 in 100% concordance with reported menarche in Tromsø 3 (that is: the same year was stated). In the oldest age group (55–73 years), 63.8% answered with perfect concordance. All age groups combined, 62.4% reported the same age at menarche, and 5.4% reported menarcheal age with a difference of more than 1 year in Tromsø 4 compared to Tromsø 3. The highest percentage of women who reported menarcheal age with more than 1-year difference (in either direction) (6.9%) was in the youngest age group (25–34 years), and it was lowest (4.6%) in women aged 55–73. We noted that 0.7% of the women reported age at menarche with more than 2 years difference.

**Table 2:** The reproducibility of self-reported age at menarche according to age in Tromsø 4 among 6731 women participating in Tromsø 3 (1986–87) and Tromsø 4 (1994–95). The percentages of the women who reported age at menarche at the same age, or with a difference of one, two or more than 2 years. The Tromsø Study

Age (Tromsø 4)	Difference in age (years) at menarche reported in Tromsø 3 and Tromsø 4				
	0 years	1 years	2 years	> 2 years	Total
25–34	58.9	34.3	6.0	0.9	100
34–44	61.9	32.7	4.9	0.6	100
45–54	63.6	31.2	4.4	0.8	100
55–73	63.8	31.7	3.8	0.8	100
Total	62.4	32.3	4.7	0.7	100

**Fig. 1:**
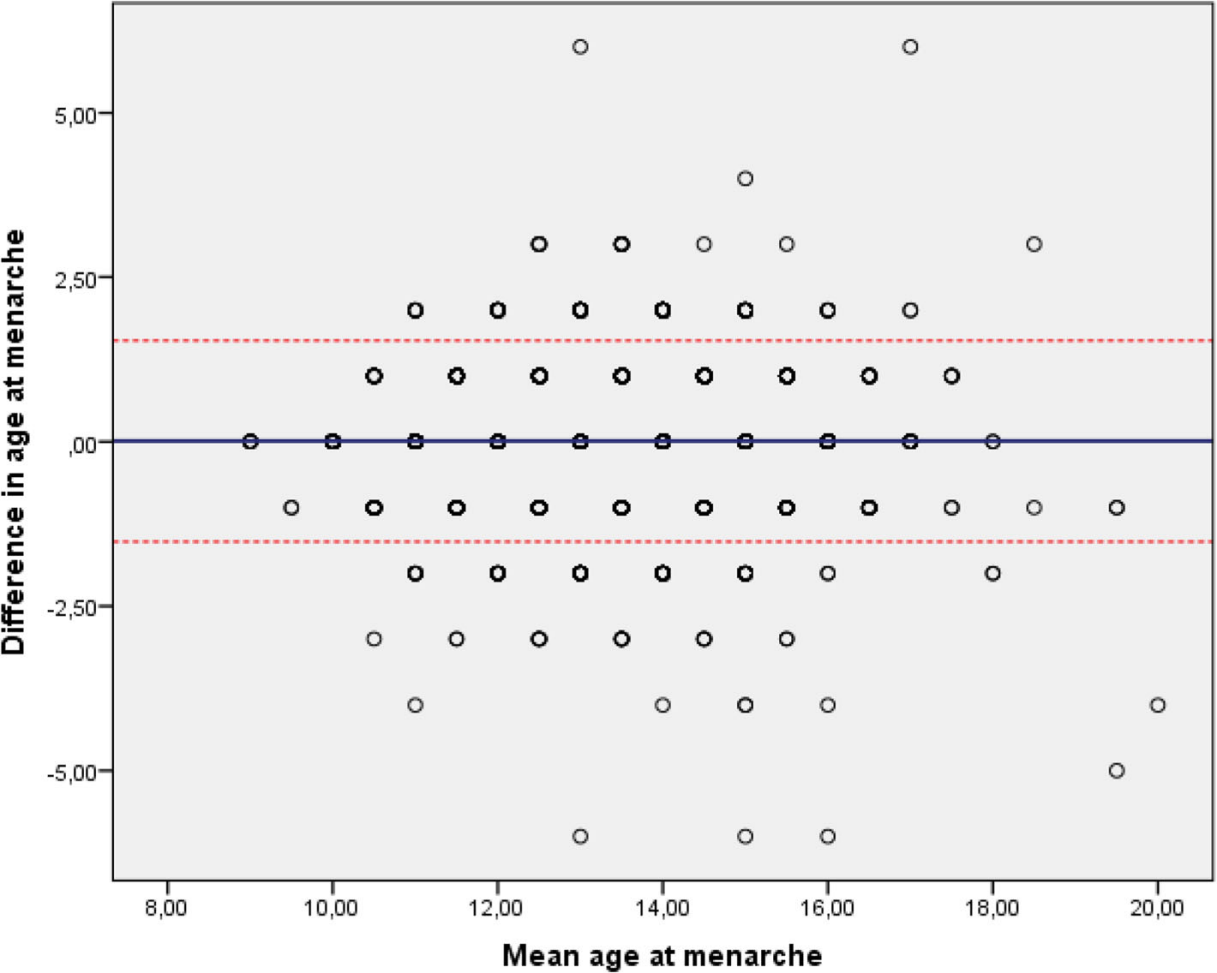
Bland-Altman plot of the agreement in self-reported age at menarche among 6731 women who attended both Tromsø 3 (1986–87) and Tromsø 4 (1994–95). The Tromsø Study. The *red dotted lines* present the limits of agreement; −1.52 – 1.54 years, the thick *blue line* in the middle represents the mean difference between reported age at menarche in Tromsø 3 and Tromsø 4. Note that one dot may represent many observations

The Fig. 1 shows the agreement between self-reported menarcheal age in Tromsø 3 and Tromsø 4 illustrated by the Bland-Altman plot. The thick blue line in the middle represents the mean difference between reported menarcheal ages at two points in time (which is 0.01 years, as stated above). The dotted red lines show the limits of agreement (the mean difference ± 1.96 SD). The limits of agreement were from −1.52 – 1.54 years, meaning that approximately 95% of the women will lie within these limits when it comes to reporting different age at menarche at two points in time. Furthermore, we note that there was only a weak correlation (*r* = 0.1) between the absolute difference and the mean age at menarche, demonstrating that the difference in age at menarche stated in Tromsø 3 and Tromsø 4 was hardly influenced by age at menarche.

The outliers observed in the plot are the women who reported menarcheal age with a high discrepancy between Tromsø 3 and Tromsø 4. Only five women reported menarcheal age in Tromsø 4 with a 6-year difference from that reported in Tromsø 3. Table 3 shows a cross-tabulation of the individual responses in Tromsø 3 and Tromsø 4. There may be a tendency, where reports do not concur completely, that the women adjusted toward what might be perceived as the “normal” age at menarche. Of women who reported 12 years as age at menarche in Tromsø 3, more women (18.5%) reported 13 years than <12 years (10.4%) in Tromsø 4. On the other hand, of women who reported 14 years as age at menarche in Tromsø 3, more women (18.6%) reported 13 years rather than 15 years (14.9%) in Tromsø 4. This tendency is even clearer for those who reported 15 as their age at menarche.

**Table 3:** The reproducibility of self-reported age at menarche according to age in Tromsø 4 among 6731 women participating in Tromsø 3 (1986–87) and Tromsø 4 (1994–95). Cross-tabulation of self-reported age at menarche in Tromsø 3 (1986–87) and Tromsø 4 (1994–95). The Tromsø Study

		Reported age at menarche – Tromsø 3						
		<12	12	13	14	15	>15	Total
Reported age at menarche - Tromsø 4	<12	479	145	20	5	0	1	650
	12	126	941	283	52	7	2	1411
	13	22	258	1241	305	45	7	1878
	14	4	47	380	1002	202	20	1655
	15	0	7	64	244	442	67	824
	>15	1	0	2	28	82	200	313
	Total	632	1398	1990	1636	778	297	6731

## Discussion

This study demonstrated a strong correlation, a good agreement and no systematic differences in self-reported age at menarche between Tromsø 3 and in Tromsø 4. This correlation strengthened with increasing age of the women when they recalled the age at menarche.

Most studies support our findings of a high reproducibility of self-reported age at menarche [25–29], but to our knowledge, no other studies have included such a large study population (6731 women), with a substantial range in the age groups included (25–73 years), and with such a long time span (7 years) between the collections of information concerning age at menarche.

We were able to investigate the correlation of reported menarcheal age between different age groups, 25 to 73 year old women. One might hypothesize that the quality of the recall of menarcheal age could decrease with increased age due to the length of time since the event occurred, as observed in a study which investigated the validity of self-reported age at menarche [19]. There were, however, no indications that reproducibility, in terms of absolute difference in age at menarche in the two surveys, the correlation coefficient (Table 1) or the proportion of the women who reported age at menarche with a difference of more than 1 year (Table 2) in older women was inferior to that of younger women.

Previous studies have tried to explain why age at menarche is well remembered and hypothesize that either menarche is remembered as an awkward, embarrassing event, or it is experienced as a “gift”, welcoming the girl to adulthood. Either way, the event in itself is an important milestone that seems to be well remembered among most females [36, 37]. One study demonstrated that the mother’s reaction to the occurrence of menarche had a large impact on how the girls experience menarche, either in a positive or negative direction [36]. A review including 14 studies focusing on the experience of menarche, summed up the 5 following concepts as important for how the girls experienced their first menstruation; how well they were prepared and their knowledge regarding the occurrence of menarche, the response from significant others, physical- and psychological experience of menarche and the socio-cultural perspective of menarche [38]. The menarcheal event seems to be a well-remembered milestone to most girls, independent of culture and religion. Both a negative and a positive association of the menarcheal occurrence would most likely be remembered and linked to an approximate place, time and age of event. This explains why both validity and reproducibility of such events normally are high. Unfortunately, we had no access to information regarding the actual age at menarche of the women (for example medical records), and therefore we cannot assess the validity of self-reported age at menarche.

We find a high reproducibility for self-reported age at menarche (a correlation coefficient of 0.84), but any less-than perfect reproducibility will give a biased (attenuated) estimate of the strength of the relationship between age at menarche and any other variable, like e.g., mortality. It has previously [2] been reported that a one-year increase in age at menarche is associated with 3% reduction in total mortality. Applying the correlation coefficient found in the present study, the “true” percentage reduction may be estimated to be approximately 4% [39].

The present study has some limitations. Menarche is a private matter, and the self-reported age at menarche could be influenced by information bias. If the menarche occurred very early or very late compared to the social circle of the woman, there is a possibility that she would rather report a menarcheal age similar to the known mean. If the women adjusted their report in this way in either Tromsø 3 or Tromsø 4, this would reduce reproducibility. This has most likely not been an important factor in our study. The age of the women included in our study and the relatively long time since they experienced menarche make it unlikely that they would intentionally misreport age at menarche. It is, however, interesting that the results in Table 3 suggest that some adjustment towards the mean or “normal” (or regression to the mean) may have taken place.

It should be noted that there were not more than 7 years between the completion of the questionnaires. It is, however, longer than in most previous studies, and it is unlikely that the women in 1994 remembered what they reported in 1987, and for most women there was a much longer time lapse (a mean of 32 and 25 years, respectively) from the actual menarche to the recall of age at menarche. Therefore, we do not believe that a longer period between giving the information would have any impact on our results. It is a limitation, though, that few older women were available for analyses. Only 1% of the 6731 women (65 women) who reported menarcheal age in Tromsø 3 and Tromsø 4 were older than 64 years of age in Tromsø 4. The oldest woman who reported menarcheal age in both Tromsø 3 and Tromsø 4 was 73 years in 1994. This woman was 66 years at the time of the Tromsø 3 survey.

Furthermore, our study would have been significantly strengthened if we had had access to medical records or other information of age at menarche that was not recalled. This would have made it possible to assess both validity and reproducibility of self-reported age at menarche. It is also a weakness that the age at menarche was reported as an integer (for example 13 years, not 13 years and 6 months). Further studies should, if possible, include data that makes it possible to assess both validity and reproducibility of self-reported age at menarche with this age reported in years and months.

The calculation of limits of agreement depends on the mean difference and the standard deviation and has the assumption that this difference is normally distributed. Outliers increase the standard deviation and therefore the limits of agreement. Excluding women with 3 or more years difference in reported age at menarche (0.7% of the women) reduced the range of limits of agreement somewhat, to – 1.38 to 1.42. Given that the data are as integers, the percentage of the women who report age at menarche with a difference of more than 1 year (5.4%) is probably a better measure of the reproducibility than the limits of agreement.

There are, however, also significant strengths. Compared to previous studies, we have included more women, we have longer periods between recording the information and a wider range of age groups available for comparison. We also believe that there is a major advantage in that the women included in our study are adults and not adolescents, which is the situation in several previous studies [22–24, 26, 31]. Our analytical strategy, including Bland-Altman plots, gives a more comprehensive evaluation of the reproducibility than an analysis restricted to the correlation coefficient alone would have given. In addition, we have been able to give the percentage of the women who reported their age at menarche with a difference of one, two or more than 2 years in Tromsø 3 and Tromsø 4 (Table 2).

The Tromsø Study is a large study, and we were able to investigate the reproducibility among 6731 women in addition to performing stratified analysis by age. We do not believe that the results are much influenced by selection bias. We have included all women who answered age at menarche in Tromsø 3 and Tromsø 4, and although attendees to population surveys differ from non-attenders [32, 40, 41], there is no reason to assume that the reproducibility of information concerning age at menarche differs between attenders and those who chose not to attend the population screenings.

## Conclusion

We found no systematic difference in self-reported age at menarche in Tromsø 3 and Tromsø 4 (7 years later), and the correlation coefficient between the reported age at menarche in the two surveys was high. The association seems to be at least as strong in older women (aged 55–73) as in younger women (aged 25–34). The limits of agreement ranged from −1.52 to 1.54, meaning that 95% of the women will report age at menarche within approximately 1.5 year between Tromsø 3 and Tromsø 4. A total of 62.4% of the women reported the exact same menarcheal age at the two points in time, and only 0.7% had more than 2-year inconsistency in self-reported menarcheal age. Our results confirm and extend the results from previous studies, confirming that self-reported age at menarche is a satisfactory measure for research purposes.

## Abbreviations

SD: Standard deviation
SPSS: Statistical package for the social sciences
